# Scene Consistency Verification Based on PatchNet

**DOI:** 10.1155/2014/298524

**Published:** 2014-07-09

**Authors:** Jinjiang Li, Xiaoqing Guo, Zhen Hua, Zhiyong An

**Affiliations:** School of Computer Science and Technology, Shandong Institute of Business and Technology, Yantai, Shandong 264005, China

## Abstract

In the real world, the object does not exist in isolation, and it always appears in a certain scene. Usually the object is fixed in a particular scene and even in special spatial location. In this paper, we propose a method for judging scene consistency effectively. Scene semantics and geometry relation play a key role. In this paper, we use PatchNet to deal with these high-level scene structures. We construct a consistent scene database, using semantic information of PatchNet to determine whether the scene is consistent. The effectiveness of the proposed algorithm is verified by a lot of experiments.

## 1. Introduction

**Figure 1:**
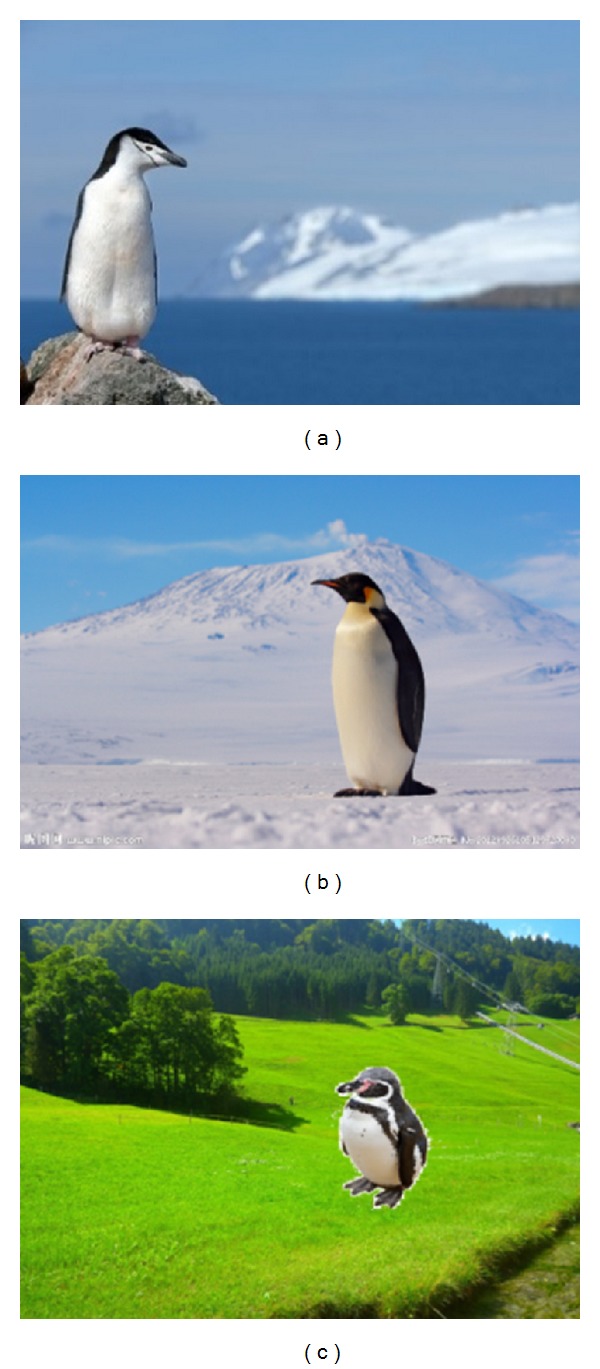
Consistent and inconsistent scenes.

Every object is in a particular scene, and the same object in different scenes will influence our perception. For example, we think that it is normal when an Antarctic penguin is in a world of ice and snow but not grassland (see Figure 1). With the development of computer network and multimedia, the technology of synthetic image is increasingly mature and is widely applied. As important media of modern information communication, digital synthetic image is developing in an unprecedented rate. How to effectively analyze, organize, and manage huge image data has been a research hotspot of multimedia technology. Among them, how to judge the consistency of image scene is a common problem in computer vision. Traditional manual classification and label management of the image have been difficult to meet the practical needs, since it will cost so much human resources and time resources. So how to employ the computer to automatically determine the scene consistency of an image becomes important.

Scene analysis [1] is one of the important research contents in image understanding, and it reflects the inclusion relation between scene and objects which has very strong cognitive structure. In some papers, they analyzed the target in the scene well to complete overall scene recognition, such as literature [2]. Researches of biology and psychology show that human visual perception will get global features of the scene firstly. We can finish scene classification without target analysis and then guide the image understanding according to the prior knowledge and local vision information. So the scene analysis provides the whole mechanism of prior knowledge for image understanding. A remarkable characteristic of human visual perception is that it can quickly grasp the expression of the meaning of a complex image; Potter [3] proved that observers can also identify the semantic category of each image by only observing a group of fast image flow through experiments. The visual and semantic information that is obtained by fast image observation (about 200 ms) is called image gist [4]. When taking pictures, the photographer always tries to put the target and features that can reflect the image gist or semantics in the center of the image. This habit makes the most similar targets for shooting have the same shooting angle in images, which means that these images have spatial similarity. For example, in many penguin images, the upper part is blue sky and under the sky is the snowcapped mountain; the penguins are standing in the snow or on the rock. That contains the context environment of the object that appears in the image.

As shown in Figure 1, obviously, we can find that the scene in (a) and (b) is very harmonious, but the scene in (c) is not consistent. But how does the computer judget? Lalonde and Efros [5] study the problem of understanding color compatibility using image composites as well as the natural images color statistics of a large dataset by looking at differences in color distribution in unrealistic and realistic images and then apply their findings to two problems which include recoloring image regions and classifying composite images. Different with literature [5], our approach has semantic information, and it is more accurate for scene identification. We first construct an image database which contains different kinds of objects (see Section 3.1). In order to simplify the algorithm, the images that we selected have clear object and are in simple background. Based on the human visual saliency features, we make saliency detection and segmentation (see Section 3.2) for the images in our database. Then we will construct the PatchNet structure for these images. For a given image, we get the salient object and its sketch (see Section 4.1). The sketch will be used for searching images that are with similar sketches in the image database, and then we will compare the contextual information (see Section 4.2) between them to identify its scene consistency.

## 2. Related Works

We need to do many works to determine whether the scene is consistent in an image, including saliency detection, image segmentation, and PatchNet constructing. So far, many people have done lots of research on related works mentioned above.

### 2.1. Visual Saliency Detection and Segmentation

In general, salient regions always keep to common criterions [6] as follows.

1. *Local Difference.* From the local perspective, salient regions always have obvious color and brightness difference with surrounding areas.
2. *Global Rarity.* From an overall point of view, characteristics of salient area are always with low frequency in the global scope. Instead, some characteristics of background region always appear frequently in the global scope region.
3. *Clustering Center.* Salient region usually has a clustered distribution center. That is to say, salient region and objects in the image have obvious characteristics of clustering, rather than dispersed.
4. *High-Level Semantics.* According to the experience of human observation, regions with some high-level features are always regions of interest, such as face recognition.

According to the above, many scholars have put forward variety of salient region detection algorithms and segmentation algorithms. Oliva and Torralba [7, 8] proposed the contextual guidance model, whose thought is that information processing has two parallel pathways: the global pathway and the local pathway. The formation of saliency map in the local pathway is mainly by extracting physical characteristics of the local scene, such as direction, brightness, and color. The highlighted area is the visual advantage region. A classical model—the Itti model [9, 10]—belongs to the local pathway. The model extracts and integrates some low-level visual features of the image. Then using two-layer neural network which is named winner-take-all, the attention points in the image are found according to decreasing sequence. It combines with multiscale image to get a saliency map and first proposed the visual attention model based on saliency for rapid analysis of the scene. The global pathway is mainly expressing the overall scene statistical information and task requirements, and it can activate the existing knowledge and experience, thereby guiding the attention.

Shi et al. [11] proposed a generic and fast computational framework called PISA (pixelwise image saliency aggregating). It is holistically complementary saliency cues based on color and structure contrasts with spatial priors. Han et al. [12] build a model for the texture, edges, and color of images by Markov random field framework and then grow saliency region by the seed value growth method in saliency map. Hou and Zhang [13] proposed a simple saliency detection method, which is independent of the object categories, object characteristics, or other forms of prior knowledge. It analyzed the logarithmic spectrum of input image, extracts spectral residual of image spectral domain, and puts forward a method to quickly establish the corresponding saliency map in spatial domain. Li et al. [14] proposed a visual saliency detection algorithm from the perspective of reconstruction errors based on the background templates. Zhu et al. [15] proposed a new method based on segmentation for saliency detection method based on multiscale super pixels. This method combined significant global and local clues. It also extracted super pixels in multiscale and displayed the normal distribution of each super pixel with its associated pixels in CIE-Lab spatial. After generating a full resolution and high quality saliency map based on significant comparison, Cheng et al. [16] employed a new iterative approach named GrabCut [17] to highlight the segmentation target. Mehrani and Veksler [18] made use of data sets in which images have been labeled by people to study and detected saliency regions. The images which have been segmented by learning classifier will be optimized through a binary graph-cut method. This algorithm also used an iterative segmentation framework, since that the manual annotation of salient regions is of low efficiency and tedious. In order to solve the problem mentioned above, Fu et al. [19] employed a saliency cut method to segment the background and target automatically. Bagon et al. [20] proposed a good image segmentation method, “component segmentation,” and it could make an image assemble itself with its own patches easily. The method produced high quality results, and it allowed patch conversion. However, looking for a good segmentation requires a complex, iterative optimization procedure. Therefore, we cannot guarantee that it can converge to a good solution. The full resolution saliency map can effectively retain the clear boundary and keep more original image frequency content than other methods. But if the most pixels in saliency regions or the background are very complex, the regions we get from detection may be background regions instead of saliency regions. In view of the characteristics mentioned above, Achanta and Süsstrunk [21] introduced a saliency region detection method. It overcame the disadvantages of full resolution methods and retained its advantages at the same time. This method utilizes the characteristics of brightness and color which is easy to be realized and has high calculation efficiency.

### 2.2. Patch Based Image Editor

Patch based method has been widely used in a variety of image and video editing tasks, such as image denoising, super resolution, image texture synthesis [22], and image stitching and image restoration [23]. Barnes et al. [23] proposed an algorithm that can quickly search for the nearest neighbor patch to match the missing part of the image. The algorithm is mainly relying on the natural correlation of images, and it allows us to find patches that match with the missing part from the image itself. Some patches which can match well with missing part can be found by random sampling. One of the advantages of patch sampling plan is that it can provide more precise control which has a great effect on image reconstruction, such as literature [24] and literature [25]. Cho et al. [25] proposed image “patch transformation” which divides the image into a number of misaligned small patches, and these patches can restructure the image. Hu et al. [26] proposed an image editing method based on PatchNet. The method divides the image into different patches based on whether the patch is a part. The location of each patch can form a contextual map. We can get the similar patch based on the comparison of contextual map.

### 2.3. Sketch and Shape Matching

Shape matching has an important application in the research of computer vision, such as image retrieval and object recognition. Researchers have done a lot of related work and proposed kinds of methods about shape matching. Zhang et al. [27] proposed a robust face sketch generation algorithm which can compose a face sketch image with many faces that from different pictures. These pictures are from more than one training set and they are in different illumination with different poses. The algorithm applies multiscale Markov random field (MRF) model for synthetizing sketch patch of local face. Klare and Jain [28] proposed a feature based face shape matching method. The method used SIFT function descriptor features directly and calculated the similarity between pairwise face sketch and match images. A combination of the two ways mentioned above is also used. Cao et al. [29] proposed an index structure and its corresponding original contour matching algorithm. They calculated the similarity of sketch and natural images. The method considers the image storage cost, the retrieval accuracy, and retrieval efficiency.

## 3. Constructing Scene Database

In order to complete the image consistency matching, we established a database which has numbers of images, saliency maps of these images, and their PatchNets. For the creation of the database, we have three steps: (1) searching for various kinds of images from the Internet (such as “Flickr”); (2) saliency extraction and segmentation of images in the database; (3) building the PatchNet of images

### 3.1. Query by “Flickr”

**Figure 2:**
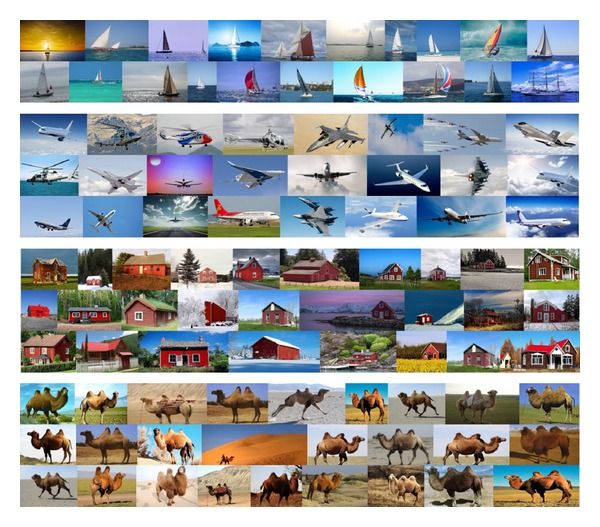
Scene dataset.

Firstly, we established an image library which contains 6000 images with different kinds of scenes and objects. We used about 60 words (such like “zebra,” “penguin,” “desert,” and “surfing”) to grab images from the “Flickr”; then we strictly screen these images in order to make it more in-line with our classification. Part of the image database can be seen in Figure 2. For these images, we will make saliency detection and segmentation further (see Section 3.2) then construct the PatchNet (see Section 3.3).

### 3.2. Salient Region Extraction and Cut

**Figure 3:**
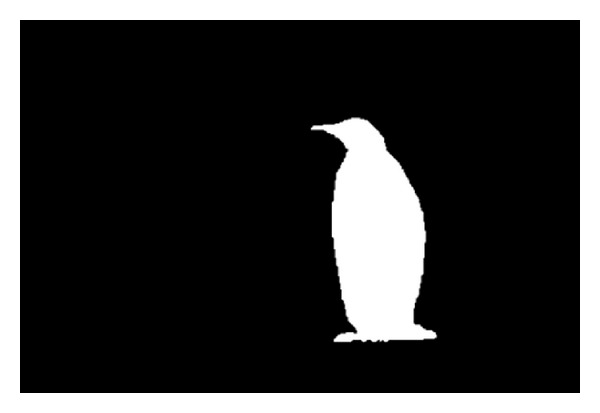
Salient region extraction.

In Section 2, we introduced some methods about image saliency detection, including the content-based bottom-to-up method. Seeing that the salient region segmentation algorithm has become mature, we do not have our algorithm. After comparing different kinds of algorithms, we decided to use a saliency detection method that is based on the global contrast [30]. The result can be seen in Figure 3; the mask is the salient region of Figure 1(b). The algorithm extracted saliency region based on regional contrast and evaluated the global difference of the image and the space consistency at the same time. It generated full resolution image easily and efficiently. We will introduce the algorithm simply as follows.

First we use graph-based image segmentation method [31] to divide the image into several regions *r*_*i*_, and we express the value of salient region by the following equation:

(1) S(rk)=∑rk≠riw(ri)Dr(rk,ri),

where *w*(*r*_*i*_) refers to the weight of the region *r*_*i*_, the value of the weight is the number of pixels in the region, and *D*_*r*_(·, ·) refers to the color distance between two regions that are defined as follows:

(2) $$\begin{array}{c} D_{r}\left(r_{1},r_{2}\right)=\overset{n_{1}}{\underset{i=1}{\sum }}\;\overset{n_{2}}{\underset{j=1}{\sum }}f\left(c_{1,i}\right)f\left(c_{2,j}\right)D\left(c_{1,i},c_{2,j}\right). \end{array}$$

In order to increase the influence between regions that have close relationship and correspondingly reduce influence between nonclose regions, literature [30] introduces a spatial weight for formula (1), which is defined as follows:

(3) $$\begin{array}{c} S\left(r_{k}\right)=\sum_{r_{k}\neq r_{i}}\exp\left(-\frac{D_{s}\left(r_{k},r_{i}\right)}{\sigma_{S}^{2}}\right)w\left(r_{i}\right)D_{r}\left(r_{k},r_{i}\right), \end{array}$$

where *D*_*s*_(*r*_*k*_, *r*_*i*_) refers to the spatial distance between two regions and *σ*_*S*_ affects the space weight. The bigger *σ*_*S*_ is, the farther region will do more contribution to the saliency of the current region. The Euclidean distance between two centroids of the two regions will be used to express the spatial distance. Here, we normalized pixel coordinates to [0, 1], and let *σ*_*S*_^2^ = 0.4.

For a color image *I*≔{*I*_*i*_}, *I*_*i*_ is the pixel in RGB color space. We want to segment image *I* which means that we need to determine the opacity value *α*≔{*α*_*i*_} of each corresponding pixel. In order to realize unsupervised segmentation, we can construct a gauss mixture model (GMM) to distinguish the color distribution of foreground and background. This can avoid setting thresholds manually and extracting binary mask directly [32]. We use the GrabCut [17] to solve the problem of image segmentation. The optimization of the Gibbs energy function is shown as follows:

(4) $$\begin{array}{c} \underset{\alpha }{\min\;}E\left(\alpha ,G,I\right)=\underset{\alpha }{\min}\left(U\left(\alpha ,G,I\right)+V\left(\alpha ,I\right)\right), \end{array}$$

where *U*(*α*, *G*, *I*) evaluates the adapted value of opacity value *α* with respect to data *I* that under *G*. *V*(*α*, *I*) evaluates the smoothness of *α*. When *α* = 1, it represents the foreground, and when *α* = 0, it represents the background. More details can be gotten from literature [17].

### 3.3. Building a PatchNet

In order to make the image editing more time saving and more close to reality, Hu et al. [26] proposed a new interactive image editing method based on database. The method can effectively express the location relationship between the representative patch and the other patches in the image to realize the image editing. In this paper, we applied it to the scene consistency detection and obtained good results.

The construction of PatchNet consists of three main steps: (1) determining the representative patch; (2) determining the real nodes and its corresponding image region; (3) forming the contextual map.

#### 3.3.1. Finding Representative Patches

**Figure 4:**
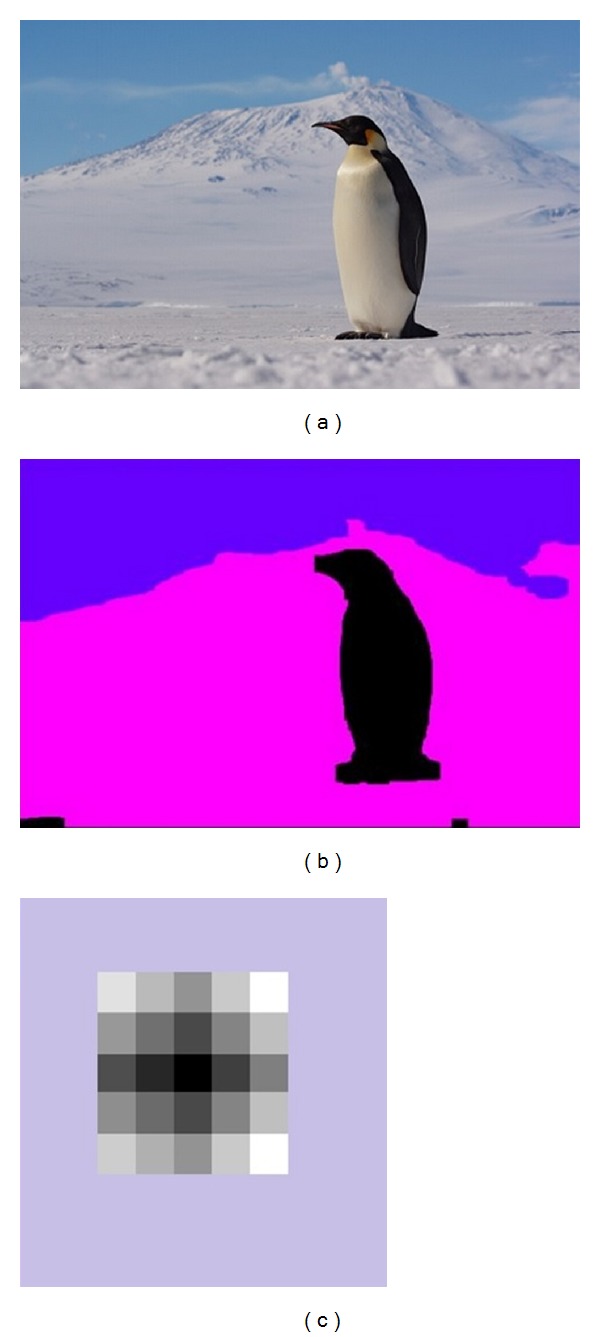
Nodes and contextual map.

Since patches of natural image are fundamental elements for visual pattern modeling and recognition, Lin et al. [33] proposed an approach for representing and recognizing objects with a massive number of local image patches. But the patches are something different from the representative patches we mentioned. In simple terms, a representative patch is a patch that can represent a coherent region of the image. For instance, in Figure 4(a), the blue sky is a representative patch. Each patch is denoted by *P* which is associated with the mask that is denoted by “*m*.” In Figure 4(b), each region in different color can be seen as an “*m*” and each mask may overlap with each other and may contain disjoint parts. The algorithm in literature [26] using a pixelwise occupies map *Q* to mark pixels that are unoccupied by any masks.

The pixels of the image are all marked unoccupied in *Q* at first, and we will iteratively perform the following steps before all pixels are occupied.

(1) Choose the pixel location *x* from all pixels that are unoccupied in *Q*, while it has the minimal gradient magnitude, and then take it as the center of the patch *P*_*x*_. Before handling more complex regions, we have to extract regions that have relatively uniform shade firstly.
(2) Use formula (5); partially adjust and reset the center of representative patch *P*_*x*_:
  (5) $$\begin{array}{c} x_{\text{new}}=\frac{\sum_{z\in P_{x}}^{}g_{z}z}{\sum_{z\in P_{x}}^{}g_{z}}, \end{array}$$
  where *z* is the pixel position of patch *P*_*x*_, *g*_*z*_ is the gradient magnitude of pixel *z*, and *x*_new_ generally is the local gradient centroid.
(3) Seek out all the image patches which can be represented by *P*_*x*_ and indicated by mask *m*_*x*_. For every pixel *y*, calculate *d*_*c*_(*P*_*x*_, *P*_*y*_) which represents the *L*_2_ norm color difference between *P*_*x*_ and *P*_*y*_. Supposing that *d*_*c*_(*P*_*x*_, *P*_*y*_) < *δ*_*x*,*y*_, then merge *P*_*y*_ into *m*_*x*_ and mark *y* that has been occupied by *Q*. *δ*_*x*,*y*_ is defined as follows:
  (6) $$\begin{array}{c} \delta_{x,y}=k{\left(\frac{\bar{g}\left(x\right)}{C\left(x,y\right)}\right)}^{m}, \end{array}$$
  where *k* is 2 and *m* is 0.5; $\bar{g}(x)$ refers to the average gradient value of patch *P*_*x*_; *C*(*x*, *y*) is the value of the average color difference between *P*_*x*_ and *P*_*y*_; and the range is [0,255]. Intuitively, the stronger the gradient of *P*_*x*_ is, the larger the threshold is (i.e., $\bar{g}(x)$ is large), so the growth limit in strong texture region is relatively not so strict. Meanwhile, if there is a relatively uniform color in *P*_*x*_, the value of the threshold mainly depends on the color difference between *P*_*x*_ and *P*_*y*_ to avoid different color regions being merged.

#### 3.3.2. Determining Real Nodes

**Figure 5:**
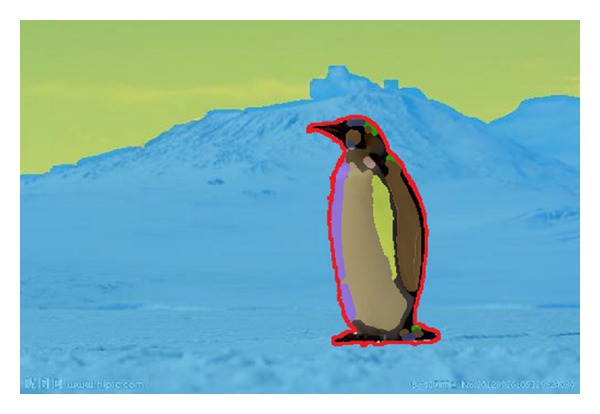
Real and compound node.

A node can represent a region of the image and nodes can be divided into real nodes and compound nodes. A real node is indivisible and a compound node can be divided into real nodes or compound nodes. For instance, in Figure 5, yellow and blue areas are indivisible and they are real nodes; the region surrounded by red border is compound nodes. According to Finding Representative Patches, we can find a series of representative patches *P*_*i*_ and each with a mask *m*_*i*_. Then do mask merging or something else to get real nods *N*_*i*_^*r*^ and each of it has a single, nonoverlap image region *Υ*_*i*_ correspondingly, and these can be represented well by *P*_*i*_.

#### 3.3.3. Graph Construction

Firstly, the real nodes in the top level are found. We can evaluate the visual dominance according to the size of the region; if the maximum region is larger than the threshold, we may put its represented patch *P*_*i*_ into the top of the image. Then the remaining nodes are divided into groups according to the spatial connectivity and the nodes in each group compose a compound node. If the biggest region is too small, then it cannot meet the standard of PatchNet and we give up this image. After finishing the steps above, we gradually expand compound node *N*_*i*_^*C*^ at each level through observing all the nodes that belong to the region it represents. Among these nodes, those who have direct contact with brother nodes will be seen as child nodes. Others can form groups according to the spatial connectivity again, and each group correspondingly has compound child nodes. We process this procedure iteratively until no more compound nodes can be found in the deeper layer. After determining the hierarchical structure, we add an edge line between each of the paired nodes *N*_*a*_ and *N*_*b*_ since they are neighbor nodes in the same level and then calculate the 5∗5 contextual map *M*(*N*_*a*_ *N*_*b*_). In *M*(*N*_*a*_ *N*_*b*_), in order to compute the pixel value of (*i*, *j*), we can count the number of pixels that *N*_*b*_ contains and these pixels are on the positions with an offset of (*i* − 2, *j* − 2) to all the pixels in *N*_*a*_. At last, normalize the map into [0,255].

## 4. Scene Consistency Verification

For an image, we will complete the image scene consistency verification in two steps: (1) detecting and segmenting the salient region of the image and finding similar shape from the database by using sketch match method; (2) constructing the PatchNet of the image and getting the contextual map to do related matching work so that we can determine whether the image scene is consistent.

### 4.1. Sketch Based Retrieval

The work of this part is inspired by Hirata and Kato [34]. In order to complete the image retrieval, they let users use sketching method to match images in database and that is sketch based image retrieval (SBIR). Cheng et al. [32] provided a simple and effective method for SBIR which is realized by a cascade filter for image retrieval.

For a given image, we first use the algorithm in Section 3.2 to get the saliency map and its boundary; the user will input the sketch and then we use a cascade way to finish later works. For each metric, we sort the similar shapes in a descending way according to the user sketches and retain the ones with higher proportion as candidate image. For example, we may retain images that keep *T*_*C*_ = 80%, *T*_*S*_ = 80%, and *T*_*F*_ = 70% according to the circularity [35], the solidity [36], and the Fourier descriptor [37], respectively. The corresponding values of these descriptors are 1, 1, and 15. The Euclidean distance with corresponding features of the user sketch is used to compare these descriptors. At last, we will sort these images by shape context [38] and choose the 20 leading images.

### 4.2. Contextual Subgraph Matching

What we did in this part benefit from “contextual subgraph matching” which is proposed by Hu et al. [26]. We denoted the input image by “a” and we can obtain its PatchNet Ψ_*a*_ according to Section 3.2, and its salient region is Ω_*a*_. We express this region as a new node *N*_*a*_ and the nodes will be denoted by *N*_*a*,*i*_^*s*^  (*i* = 1, 2,…, *L*) that are in the same level with *N*_*a*_, where *L* is the total number of nodes that are at the same level with *N*_*a*_, and we defined these nodes as the contextual environment of node *N*_*a*_. In this paper, we will match “*a*” with images that retrieved from our database one by one before we get an image whose scene is consistent with “*a*” or the images we retrieved from database have been matched all. Denote the image of database by “*b*”, its PatchNet by Ψ_*b*_, the salient region which is similar to Ω_*a*_ is Ω_*b*_, and its corresponding node by *N*_*b*_. Also, we defined the nodes as *N*_*b*,*j*_^*s*^  (*j* = 1, 2,…, *K*, *K* may  not  be  equal  to *L*).

Defining the distance *D*(*N*_*a*,*i*_^*s*^, *N*_*b*,*j*_^*s*^) reasonably is critical to scene consistency verification and in the definition of the distance, there are two kinds of very important similarities: one is the appearance similarity between *P*(*N*_*a*,*i*_^*s*^) and *P*(*N*_*b*,*j*_^*s*^), it means that the two nodes have the same representative patches; the other is the location similarity between (*N*_*a*_, *N*_*a*,*i*_^*s*^) and (*N*_*b*_, *N*_*b*,*j*_^*s*^). If there may be some overlap between the contextual maps *M*(*N*_*a*_, *N*_*a*,*i*_^*s*^) and *M*(*N*_*b*_, *N*_*b*,*j*_^*s*^), which means that if *N*_*a*_ is something aligned with *N*_*b*_ spatially, then there may be similarities between *N*_*a*,*i*_^*s*^ and *N*_*b*,*j*_^*s*^. This is because different images have different structure, even though they may be under the same scene. For instance, in an image the sky may be at the upper left of a house, but in another image the sky is at the upper right of the house. For the two images, although they could match very well, strict similarity measure may give a low matching score. Avoiding this, a more flexible contextual overlap is proposed as follows:

(7) $$\begin{array}{c} O\left(N_{a,i}^{s},N_{b,j}^{s}\right)=\sum \left(M\left(N_{a},N_{a,i}^{s}\right)\cdot M\left(N_{b},N_{b,j}^{s}\right)\right). \end{array}$$

This is the two maps' dot product sum. When the two maps share common high probability regions, the overlap will be very high and this makes it more flexible to match those images whose structure is slightly different.

Definition of the overall distance between the two nodes is as follows:

(8) $$\begin{array}{c} D\left(N_{a,i}^{s},N_{b,j}^{s}\right)=\{\begin{array}{cc} d_{c}\left(P\left(N_{a,i}^{s}\right),P\left(N_{b,j}^{s}\right)\right), & O\left(N,N\right)\geq T_{o}\\ \infty , & \text{else}, \end{array} \end{array}$$

where *d*_*c*_(*P*(*N*_*a*,*i*_^*s*^), *P*(*N*_*b*,*j*_^*s*^)) is the difference of patch appearance, unless the contextual overlap between the two nodes is no bigger than the threshold value *T*_*o*_ (here it is set to 10), the two nodes are treated incompatible, and we set the distance to infinity, else their similarities are the distance of the appearance distance between patches. If there is an image that can make the appearance distance of two nodes less than the threshold (in this paper we set it to 900), then we think that the image scene is consistent, else not consistent.

## 5. Experimental Results

In order to demonstrate the feasibility and effectiveness of the algorithm in this paper, we implement the experiment on a PC with an Intel Corei3 CPU and 4 GB RAM, running 64 bit Windows 7. For the simplicity of computation, the length and the width of images in our database are normalized to no more than 800 pixels. Now we will detail the experiment results as follows.

### 5.1. Results of Saliency Detection and Their Corresponding PatchNet Appearance Map

**Figure 6:**
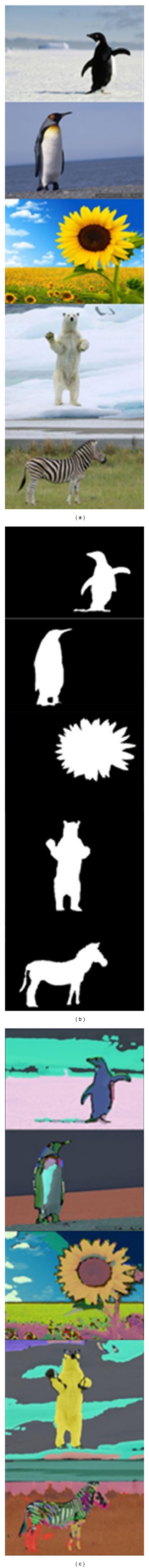
Saliency and PatchNet appearance map: original image (a), mask (b), and PatchNet appearance map (c).

Since there are so many images in our database, we will only show part of the results. See Figure 6; the left column is the original image; the middle column is the mask of saliency map which also can be seen as the sketch of object; and the right column is the PatchNet appearance map. We can see the background and salient object from Figure 6.

### 5.2. Results of Sketch Matching

**Figure 7:**
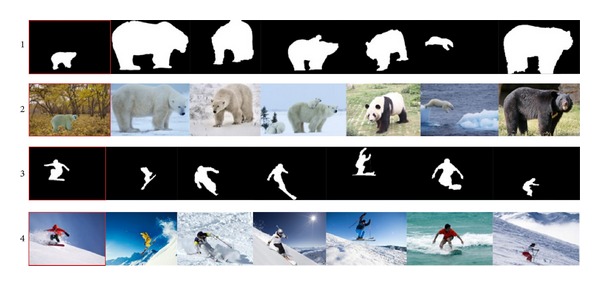
Matching results.

By using the method of sketch based image retrieval, we try to get the similar sketches from the database according to input mask and then get the images whose masks are similar to the input mask. In Figure 7, the first and the third rows with red border are our input image mask; the rest is the searching results. The second and the fourth rows are the original images that correspond to the masks of first and the third rows. We can see part of the matching results in Figure 7.

### 5.3. Results of Scene Consistency Verification

**Figure 8:**
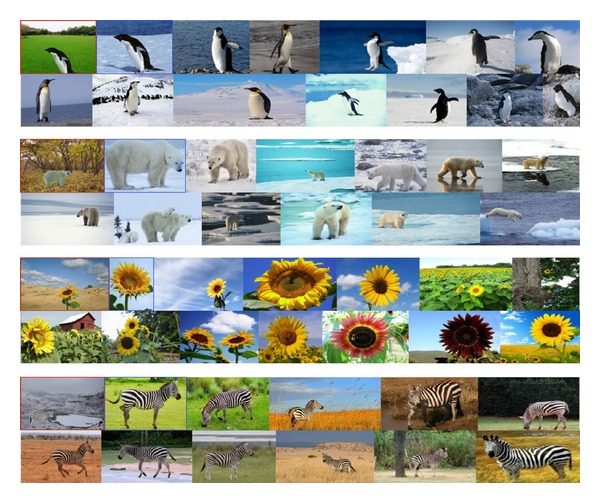
Results of scene consistency verification.

After obtaining the matching results, we will complete the contextual map and then judge whether the scene is consistent. Looking at Figure 8, the images with red borders are that scene is inconsistent, and the ones with blue borders are that scene is consistent. The other images are part of the ones that are from our database who have been matched with the input image. Experimental results demonstrate the feasibility and effectiveness of our algorithm.

**Figure 9:**
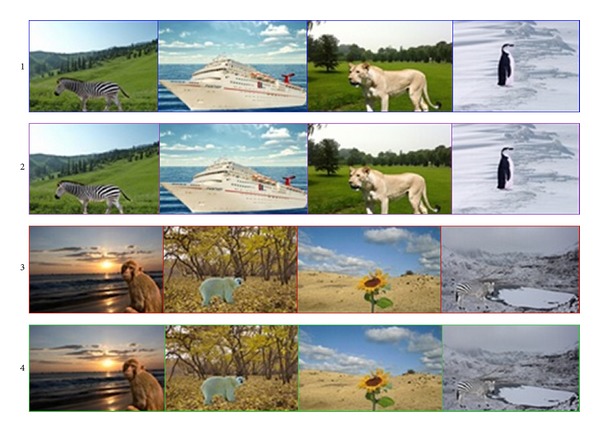
Results of scene consistency verification. Row 1 and row 3 are results of our experiments and row 2 and row 4 are results of literature [5]. The border color indicates the verification result for each image (blue and green: scene is consistent; red and purple: scene is inconsistent).

**Figure 10:**
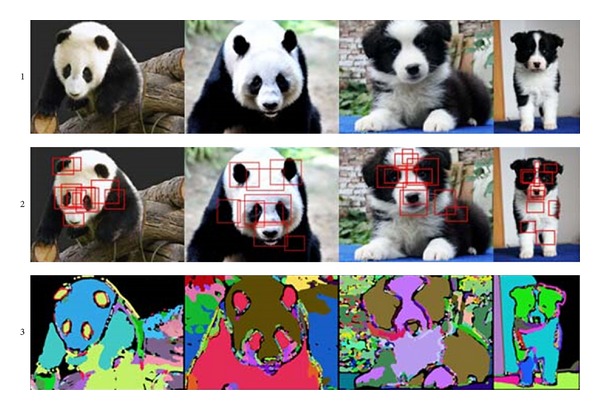
Discriminative patterns and PatchNet map.

In order to show the effectiveness of the method in our paper, we will compare the method in our paper with the methods of literature [5] (classifying composite images into realistic versus nonrealistic) and literature [39] (scene identification), respectively (just look at Figures 9 and 10). The method of literature [5] is just using the color compatibility for assessing image realism. The method of literature [39] proposed a discriminative measure to rank image patterns sampled from target scene classes for scene identification. The advantage of our algorithm is that we use the contextual information of the image.

In Figure 9, the images with blue borders are consistent scene and the ones with red borders are inconsistent in our experiments. And the images with green borders are those whose scenes are consistent and the ones with purple borders are inconsistent in literature [5]. From Figure 9 we can find that some scenes are consistent (e.g., the images in row 2), but it may be judged as unrealistic images in literature [5]. Also, some scenes are obviously inconsistent (e.g., the images in row 4) but literature [5] judged them as realistic images since the color of the objects in those images is something like the background. Certainly we may get the wrong result (e.g., the images in row 1, but the scenes are really consistent).

In Figure 10, row 1 is input images; row 2 is the results of literature [39]; and row 3 is PatchNet appearance map. From Figure 10, we can see that the objects (pandas and dogs) have similar discriminative patterns (see row 2 in Figure 10) and will be judged as the same class. So naturally the scenes are identified the same as in literature [39]. Obviously pandas and dogs are of neither the same class nor the scenes. Seen from the PatchNet appearance map (row 3 in Figure 10), although the discriminative patterns of the pandas and the dogs are very similar, the semantic of representative patch is different.

To compare the accuracy of our method with literature [5] (Table 1), we selected 6 classes from our database, and each class has 20 images. Also we add some inconsistent images and some irreal but consistent images for each class. For class penguin, there are 24 consistent scene images and only 20 real images. The recall rate of the two methods is compared. Some irreal but consistent images are judged as inconsistent scene, so the recall rate for literature [5] is low.

## 6. Conclusions

In this paper, we propose a new method to judge the scene consistency. We construct a consistent scene database with minimal manual intervention. By downloading, saliency detecting, segmentation, matting, and analyzing amounts of different scene images from the Internet, we use semantic information of PatchNet to determine whether or not the scene is consistent. PatchNet summarizes image appearance in terms of a small number of representative patches for image regions, linking them in a hierarchical graph model to describe image structure. Fast scene semantic matching can be achieved by graph matching. For existing scene image in our semantic-based scene database, it is accurate to judge scene consistency. For nonexistent scene image, using our pipeline, it is easy to construct a new class of scene image. We achieved a good result in a certain extent but still have a long way to go for wide applications. In the future we still have a lot to do, including the following.

1. The algorithm of saliency detection needs to be improved in order to reduce the computing time consumption. A good saliency detection algorithm is beneficial to improve the accuracy of scene classification.
2. In this paper we use static images as research object, but with the development of science and technology, composite video may be coming into people's daily life. For example, some films have a lot of postprocessing. If the scene is not consistent, it will bring unexpected visual to the audience. So the scene consistency detection applied to video field may be a significant research direction.
3. The coverage of the database in this paper is still limited, and our experiment only proved the effectiveness of part images. So next, we need to enrich our database and expand the application.

## Figures and Tables

**Table 1:** The accuracy of our method and the method of literature [5].

	Penguin	Ship	Skating	Panda	Sunflower	Monkey
Inconsistent	5	4	5	6	4	5
Irreal but consistent	4	4	3	5	6	5
Recall of ours	0.92	0.88	0.91	0.88	0.96	0.92
Recall of [5]	0.71	0.67	0.74	0.68	0.73	0.72
